# EGFR-TKIs与二甲双胍联合治疗在非小细胞肺癌EGFR-TKIs获得性耐药中的研究进展

**DOI:** 10.3779/j.issn.1009-3419.2023.106.22

**Published:** 2023-11-20

**Authors:** Jiamin WANG, Pan LIU, Lisha YING, Rui ZHU, Chaodan YANG, Ying YANG, Dan SU

**Affiliations:** ^1^310024 杭州，中国科学院大学杭州高等研究院分子医学院; ^1^School of Molecular Medicine, Hangzhou Institute for Advanced Study, University of Chinese Academy of Sciences, Hangzhou 310024, China; ^2^201203 上海，中国科学院上海药物研究所; ^2^Shanghai Institute of Materia Medica, Chinese Academy of Sciences, Shanghai 201203, China; ^3^100049 北京，中国科学院大学; ^3^University of Chinese Academy of Sciences, Beijing 100049, China; ^4^310022 杭州，浙江省肿瘤医院病理科，中国科学院杭州医学研究所; ^4^Department of Pathology, Zhejiang Cancer Hospital, Hangzhou Institute of Medicine, Chinese Academy of Sciences, Hangzhou 310022, China; ^5^310053 杭州，浙江中医药大学第二临床医学院; ^5^The Second Clinical Medical College, Zhejiang Chinese Medicine University, Hangzhou 310053, China

**Keywords:** 表皮生长因子受体酪氨酸激酶抑制剂, 二甲双胍, 靶向治疗, 肺肿瘤, 联合治疗, Epidermal growth factor receptor-tyrosine kinase inhibitors, Metformin, Targeted therapy, Lung neoplasms, Combined therapy

## Abstract

靶向表皮生长因子受体（epidermal growth factor receptor, EGFR）的EGFR酪氨酸激酶抑制剂（EGFR-tyrosine kinase inhibitors, EGFR-TKIs）对EGFR突变阳性的非小细胞肺癌（non-small cell lung cancer, NSCLC）患者具有较好的疗效，但是会不可避免地出现耐药问题。随着个体化和联合治疗的应用和拓展，越来越多的研究显示二甲双胍联合给药在临床治疗中有效解决了EGFR-TKIs获得性耐药的难题，并延长了NSCLC患者的生存期。EGFR-TKIs联合二甲双胍有望成为NSCLC患者出现EGFR-TKIs耐药后可选择的治疗方法，本文拟对EGFR-TKIs与二甲双胍联合治疗NSCLC EGFR-TKIs获得性耐药中的研究进展进行总结，以期为出现EGFR-TKIs获得性耐药的NSCLC患者的治疗提供新的思路。

## Abstract (en)

Epidermal growth factor receptor-tyrosine kinase inhibitors (EGFR-TKIs) targeting EGFR are effective in EGFR mutation-positive non-small cell lung cancer (NSCLC) patients, but drug resistance is inevitable. With the application and expansion of individualized and combined therapy, more and more studies have shown that combined administration of Metformin effectively solves the problem of acquired drug resistance to EGFR-TKIs in clinical treatment and prolongs the survival of patients with NSCLC. EGFR-TKIs combined with Metformin is expected to be the treatment method of choice for NSCLC patients with EGFR-TKIs resistance. This paper intends to summarize the research progress of EGFR-TKIs combined with Metformin in the treatment of EGFR-TKIs acquired resistance in NSCLC, in order to provide a new idea for the treatment of NSCLC patients with acquired resistance to EGFR-TKIs.

肺癌是最常见的恶性肿瘤之一，其中非小细胞肺癌（non-small cell lung cancer, NSCLC）占所有肺癌的85%。目前NSCLC的治疗除常规手术和放化疗外，靶向治疗已为越来越多的患者所选用^[1]^。表皮生长因子受体（epidermal growth factor receptor, EGFR）是NSCLC患者最常见的驱动基因。前期EGFR-酪氨酸激酶抑制剂（EGFR-tyrosine kinase inhibitors, EGFR-TKIs）靶向药物的使用对延长NSCLC患者的寿命起到了明显的作用。它通过与细胞内酪氨酸激酶（tyrosine kinases, TKs）的腺嘌呤核苷三磷酸（adenosine triphosphate, ATP）结合部位特异性结合，降低EGFR自磷酸化，抑制EGFR信号传导，从而抑制细胞增殖、阻断细胞周期进程、刺激细胞凋亡^[2]^。第一代小分子EGFR-TKIs，包括吉非替尼、厄洛替尼和艾替尼，以竞争和可逆的方式与EGFR结合。而以阿法替尼和达可替尼为代表的第二代EGFR-TKIs被认为是EGFR和Erb B酪氨酸激酶受体家族（Erb B2、Erb B3和Erb B4）其他成员的不可逆共价抑制剂。然而，虽然第三代EGFR-TKIs奥希替尼、阿美替尼和泽拉替尼既靶向EGFR敏感突变又靶向T790M耐药突变，但与其他靶向治疗一样，大部分患者在使用药物之后的4-24个月内，都会出现耐药的情况^[3]^。总的来说，EGFR-TKIs的更迭提升了疗效并扩大了适应人群的范围，但通常在用药10-24个月后，就会面临获得性耐药问题，这使得EGFR-TKIs在NSCLC临床治疗中的应用受到了很大的限制^[4]^。

二甲双胍源于山羊豆，是一种具有抑制肝脏糖异生、促进外周葡萄糖摄取等功效的双胍类衍生物^[5]^。研究^[6]^发现，二甲双胍不仅能控制糖尿病症状，还能降低2型糖尿病（type 2 diabetes mellitus, T2DM）患者多发恶性肿瘤的发生率，并能有效改善癌症患者的预后。近年来，研究^[7]^显示，通过使用二甲双胍联用EGFR-TKIs治疗EGFR突 变阳性NSCLC，可以有效地克服患者获得性耐药，改善患者的生存状态，提高生存率。目前EGFR-TKIs联合二甲双胍已成为临床上一种可行的预防和延缓耐药性的方法^[8]^。本文旨在探讨EGFR-TKIs联合二甲双胍治疗EGFR突变型NSCLC的研究进展，以期为该类型NSCLC患者的临床治疗提供参考。

## 1 EGFR-TKIs在NSCLC中的获得性耐药机制研究

### 1.1 上皮-间充质转化（epithelial-mesenchymal transition, EMT）

在肿瘤细胞发生获得性EGFR-TKIs耐药的过程中，往往还伴随着失去上皮表型，转化为间质表型的细胞表型变化。EMT是指上皮细胞在细胞因子、药物、物理损伤等因素的作用下，失去了上皮细胞固有的黏附性和连接性，转化为具有更好的运动性、侵袭性和抵抗调往能力的间质细胞^[9]^。Weng等^[10]^用第一代TKIs吉非替尼处理NSCLC细胞株，使其对吉非替尼获得性耐药，通过其耐药机制的研究发现，NSCLC细胞株在耐药的过程中获得了间充质表型，并且伴随着E-钙黏蛋白（E-cadherin）表达下调和波形蛋白表达增加的变化。此外，通过对亲本细胞株E-cadherin的敲除可以赋予其吉非替尼耐药性，同时增加集落形成和迁移能力。而对耐药细胞株波形蛋白敲低可以增加其对吉非替尼敏感，同时菌落形成和迁移能力也有所下降。这些研究证明在EGFR突变型NSCLC细胞中，EMT是EGFR-TKIs的获得性耐药的机制之一。因此，考虑EMT对治疗的影响在开发针对EGFR-TKIs获得性耐药的治疗方法时至关重要。

### 1.2 丝裂原活化蛋白激酶（mitogen-activated protein kinases, MAPK）通路异常

EGFR-TKIs获得性耐药的一个重要原因是MAPK相关信号通路发生异常。RAS、RAF和MEK1/2基因编码MAPK通路的关键蛋白，基因的改变可引起MAPK通路的紊乱，导致EGFR-TKIs获得性耐药。Ercan等^[11]^通过在体内和体外模型中研究不可逆酪氨酸激酶抑制剂WZ4002对EGFR T790M突变NSCLC的耐药性机制，研究发现NSCLC对WZ4002的耐药是由细胞外信号调节激酶（extracellular signal-regulated kinase, ERK）信号异常激活引起的，这是由MAPK1扩增或ERK信号负向调控导致。也就是说，通过抑制MAPK1或ERK可恢复对WZ4002的敏感性并防止耐药性的出现。此外，该研究在对第一代TKIs厄洛替尼获得性耐药的EGFR突变型NSCLC患者中进一步鉴定了MAPK1扩增。

### 1.3 激活核因子κB（nuclear factor kappa-B, NF-κB）信号途径

活化B细胞的NF-κB信号途径的激活也会引起NSCLC对EGFR-TKIs获得性耐药。Bivona等^[12]^为了探讨NF-κB通路在第一代TKIs厄洛替尼耐药细胞株中TKIs敏感性的作用，通过对NF-κB通路中的主要亚基RELA的敲除实验，发现敲除RELA可以诱导耐药细胞株中的厄洛替尼敏感性。此外，该研究发现，耐药细胞株对NF-κB抑制蛋白（inhibitor of NF-κB, IκB）的磷酸化作用明显，并通过NF-κB的敲低实验，发现耐药细胞株对厄洛替尼的部分耐药性有所显现。这些数据表明，激活NF-κB信号通路，可以部分赋予肿瘤细胞对EGFR-TKIs获得性耐药。

### 1.4 EGFR T790M突变

第一、二代EGFR-TKIs最普遍的耐药机理为EGFR 20号外显子获得性T790M突变，约50%的病例在耐药后检测到了该突变^[13]^。目前，已有奥西替尼、阿米替尼、阿维替尼等第三代TKIs被用于含有EGFR T790M突变的患者。作为与厄罗替尼或吉非替尼的结合位点的T790，位于EGFR催化区域的ATP结合点，而T790M指的是甲硫氨酸取代了苏氨酸，暴露出大量的甲硫氨酸残基，阻止EGFR-TKIs的芳香结构进入EGFR催化区域，进一步阻碍与ATP位点的结合^[14]^，进而产生抗药性。Oxnard等^[15]^通过对41例EGFR-TKIs获得性耐药的NSCLC患者进行了肿瘤活检以进行基因组分析，发现68%的患者存在T790M突变。此外，研究认为由于患者的耐药性在遗传上具有异质性，靶向治疗的有效性会因此受到损害，因此在制定耐药的策略时，需要考虑这一点。因此该研究认为EGFR T790M突变有望成为研究EGFR-TKIs获得性耐药的突破点，这同时拓宽了EGFR-TKIs获得性耐药患者的临床治疗思路。

## 2 二甲双胍在肿瘤中的应用研究

作为双胍类代表性药物的二甲双胍，除了其经济、安全、有效等优势，在相关研究中发现，对肿瘤也有一定的抑制作用，临床上应用较为广泛。二甲双胍可以激活腺苷酸活化蛋白激酶（adenosine monophosphate-activated protein kinase, AMPK）通路^[16]^、调节胰岛素样生长因子-1水平途径^[17]^、抑制肿瘤细胞迁移及转移途径^[18]^，对肿瘤细胞的凋亡、自噬、细胞周期、能量代谢、EMT和免疫等细胞生物活动进行调控^[19]^。

### 2.1 二甲双胍促进肿瘤细胞凋亡

细胞凋亡是一种独特的细胞死亡模式，在生物进化过程、细胞内环境稳定性过程和多种体系的形成中发挥着关键的作用，其主要特征是由死亡细胞主动参与并受到多种基因的调控。二甲双胍可通过抑制雷帕霉素靶蛋白（mammalian target of rapamycin, mTOR）的活性来控制细胞内的能量代谢。研究^[20]^发现二甲双胍通过干扰肿瘤微环境中的氧化应激途径，对线粒体正常功能进行干扰，使肿瘤细胞内过度积累活性氧（reactive oxygen species, ROS），从而引起肿瘤细胞凋亡。此外，Luo等^[21]^发现二甲双胍可以抑制蛋白激酶A（protein kinase A, PKA）活性，并通过激活AMPK进而活化其下游糖原合成酶激酶3β（glycogen synthase kinase-3β, GSK-3β），下调Survivin蛋白表达并诱导其降解，从而促进肺癌细胞凋亡。

### 2.2 二甲双胍诱导肿瘤细胞周期停滞

细胞周期是指细胞完成分裂和增殖的过程，是维持细胞正常生命活动的基本特征。细胞周期失调是肿瘤形成的重要原因，可导致细胞生长和分化异常，从而诱发恶性细胞增殖。Wu等^[22]^发现二甲双胍可以通过对膀胱癌细胞AMPK磷酸化激活，同时降低Yap1和CDK4/6的表达，进而诱导肿瘤细胞G_1_期阻滞，而不引起细胞凋亡，起到抑制肿瘤细胞增殖的作用。Ashinuma等^[23]^通过体外克隆形成和细胞增殖实验发现二甲双胍在肺癌细胞中存在剂量依赖性的抑制作用，并且会诱导G_0_/G_1_期积累，出现周期阻滞，从而发挥细胞毒性，抑制肺癌细胞增殖的作用。

### 2.3 抑制肿瘤细胞迁移及转移

上皮细胞源性恶性肿瘤可通过细胞形态的EMT改变、细胞骨架的改变、细胞表面标志物的改变、基因表达的改变等一系列变化获得迁移和侵袭能力，这对肿瘤的发生和转移具有重要意义^[24]^。研究^[25]^发现，低剂量（0.1 mmol/L）的二甲双胍可以降低癌细胞中survivin蛋白和Snail、Twist等EMT相关蛋白的表达，进而抑制卵巢癌细胞EMT，从而发挥抗肿瘤功效。Liu等^[26]^发现二甲双胍可通过作用于转化生长因子-β1（transforming growth factor-beta 1, TGF-β1）/转录激活因子-3（signal transducer and activator of transcription 3, STAT3）信号通路，抑制肿瘤细胞EMT，进而逆转前列腺癌细胞对恩杂鲁胺的耐药性，从而抑制癌细胞增殖。

## 3 EGFR-TKIs联合二甲双胍逆转EGFR-TKIs获得性耐药的机制研究

### 3.1 通过抑制EMT延缓或逆转EGFR-TKIs获得性耐药

研究者基于EGFR-TKIs联合二甲双胍给药的良好应用前景，开展了多项关于二甲双胍治疗EGFR-TKIs耐药的NSCLC的临床前研究，其中部分研究显示二甲双胍可以通过发挥抑制EMT的作用，延缓或逆转EGFR-TKIs耐药机制。Barrios-Bernal等^[27]^通过研究第二代TKIs阿法替尼与二甲双胍单独和联合用药两种方式对EGFR-TKIs获得性耐药的NSCLC细胞株的影响，发现联合用药通过抑制EMT转化和减少糖酵解两个过程诱导EGFR通路的抑制作用，并且比单用阿法替尼表现出更高的细胞毒性作用。而二甲双胍对非转化细胞产生肝糖异生的抑制和加速葡萄糖摄取的作用，同时对所有NSCLC细胞株诱导协同细胞毒性和促凋亡作用，这可以加剧阿法替尼的抑制作用。值得注意的是，相比于TKIs高亲和力NSCLC细胞株，TKIs低亲和力NSCLC细胞株表现出更高的协同抑制效应。Kurimoto等^[28]^通过研究携带EGFR突变的人肺腺癌细胞系中EMT诱导和逆转的变化，对EMT诱导与逆转的分子机制进行探索，发现二甲双胍可以对携带EGFR突变的人肺腺癌细胞系的EMT起到逆转的作用，与吉非替尼联用可以发挥恢复并促进细胞凋亡的作用。有趣的是，研究发现尽管EMT诱导与Smad3表达的升高有关，但其逆转与Smad3表达的减少无关，而程序性死亡配体1（programmed cell death ligand 1, PD-L1）表达在EMT诱导过程中升高，并且在逆转过程中被抑制，这个结果表明非Smad3途径参与了EMT驱动的免疫抑制。也就是说，PD-L1表达的降低可能导致携带EGFR突变的人肺腺癌细胞系中EMT发生逆转。这个研究表明联合用药可以通过促进细胞凋亡和减少PD-L1的表达，进而抑制TGF-β/成纤维细胞生长因子2（fibroblast growth factor 2, FGF-2）诱导的EMT，从而提高EGFR-TKIs获得性耐药NSCLC细胞系对TKIs药物的敏感性。而Li等^[29]^研究显示EGFR-TKIs获得性耐药性的NSCLC细胞用二甲双胍处理后，肺癌细胞对埃洛替尼或吉非替尼的敏感性增加。肺癌细胞的增殖、迁移和侵袭在联合吉非替尼和二甲双胍处理EGFR-TKIs耐药细胞后，均有明显的抑制现象。在小鼠模型中，与吉非替尼或二甲双胍单独治疗相比，联合治疗可以更有效地减少肿瘤的大小。因此，研究者把联合治疗对EGFR-TKIs获得性耐药的作用归因于联合治疗逆转了EGFR-TKIs耐药细胞中的EMT转化，抑制了白介素6（interleukin 6, IL-6）信号的激活，而这种协同作用与二甲双胍降低STAT3和AKT磷酸化的激活，逆转EMT作用有关。

以上临床前研究结果表明二甲双胍联合EGFR-TKIs可以通过抑制EMT转化的机制延缓或逆转EGFR-TKIs获得性耐药，从而改善EGFR-TKIs获得性耐药的NSCLC细胞的治疗效果。虽然这种延缓或逆转EGFR-TKIs耐药机制具有极大的潜在临床获益，但仍需要大量的临床前数据来加速这种治疗方案的临床转化，以确保其能够获得更好的临床效果。因此，研究者需要不断完善研究数据，并通过多种方式进行验证，以确保这种治疗方案能够取得最佳效果。

### 3.2 通过抑制特定信号通路延缓或逆转EGFR-TKIs获得性耐药

二甲双胍除了可以通过发挥抑制EMT的作用外，还可以通过抑制某些信号转导途径来延缓或逆转EGFR-TKIs耐药机制。Li等^[30]^通过在体外和体内用二甲双胍处理EGFR-TKIs获得性耐药的NSCLC细胞，发现二甲双胍通过激活AMPK进而抑制下游ERK/NF-κB信号转导途径，从而延迟或逆转肺癌细胞EGFR-TKIs的获得性耐药，进而对肺癌发挥抑制作用。而Morgillo等^[31]^通过在体内外对携带LKB1野生型基因的NSCLC细胞系进行二甲双胍与吉非替尼的单药和联合用药实验，发现相较于单独用药，联合用药诱导的抗增殖和促凋亡作用更加强烈。二甲双胍单药处理可以通过增加C-RAF/B-RAF异二聚作用诱导MAPK的活化和磷酸化，但是在加入吉非替尼后，可以通过对EGFR磷酸化和下游信号传导的抑制消除MAPK活化这一现象。这些结果表明，二甲双胍和吉非替尼联合用药可以通过RAS/RAF/MAPK途径同时对EGFR-TKIs获得性耐药的NSCLC细胞系发挥抑制增殖和促凋亡作用。Pan等^[32]^从亲本细胞系中建立了罗西替尼抗性细胞系，发现其对罗西替尼的抗性比亲本细胞高出90倍。此外，他们发现在抗性细胞中，活化B细胞的NF-κB信号、分子蛋白p50、p65亚基及其抑制蛋白IKBa、IKKa/β的磷酸化水平均高于亲代细胞。二甲双胍通过减少细胞增殖和侵袭，克服了EGFR-TKIs抗性细胞系对罗西替尼的获得性耐药。也就是说，二甲双胍联合罗西替尼可以协同抑制EGFR-TKIs抗性细胞系中p-IKBa、p-IKKα/β、p50和p65信号。综上所述，二甲双胍可以通过抑制NF-κB信号传导使耐药细胞对EGFR-TKIs一线药物罗西替尼重新敏感。

这些临床前数据表明，EGFR-TKIs和二甲双胍联合治疗具有良好的治疗前景，可以通过协同抑制某些相关信号通路不同程度地延缓或者逆转EGFR-TKIs耐药性，从而提高治疗效果。这一发现为我们提供了一个新的视角，可以帮助我们更好地从联合药物与信号通路的相互作用关系来理解EGFR-TKIs耐药性的机理，从而对应用于NSCLC的临床治疗方法提供参考，但仍需通过大量的临床前试验，优化最有潜力的靶向治疗方案，而具体哪种联合治疗方案可以获得最大的临床获益，还需要不断探索。

## 4 EGFR-TKIs联合二甲双胍治疗EGFR-TKIs获得性耐药的临床研究

临床前研究数据揭露了EGFR-TKIs联合二甲双胍在延缓或者克服获得性耐药中的作用机制。据此，研究者针对EGFR-TKIs联合二甲双胍治疗EGFR-TKIs获得性耐药开展了多项临床实验。NCT03071705^[33]^是一项开放标签、随机的I/II期临床研究，也是首次前瞻性地评估EGFR-TKIs联合二甲双胍应用在携带EGFR突变的晚期NSCLC患者的研究。该研究纳入具有激活EGFR突变的IIIB-IV期肺腺癌患者，主要目的是对第一代EGFR-TKIs厄洛替尼单药和联合二甲双胍治疗患者的无进展生存期（progression-free survival, PFS）进行评估。研究发现，与单独接受EGFR-TKIs治疗的患者相比，EGFR-TKIs联合二甲双胍治疗的患者PFS和总生存期（overall survival, OS）明显延长，客观缓解率（objective response rate, ORR）显著提升；另外，研究表明二甲双胍治疗与较长的PFS和OS独立相关，而不会显著增加腹泻等不良事件。值得注意的是，这项I/II期临床研究成果为后续设计更大规模的双盲、安慰剂对照的III期临床试验提供了有力的依据，也为NSCLC患者临床药物联合治疗方法提供了参考。而Hung等^[34]^基于一个全面的医疗保健数据库——健康保险研究数据库，探讨EGFR-TKIs治疗的糖尿病合并NSCLC患者中二甲双胍的效用，目的是提供临床治疗策略的参考。通过研究二甲双胍在接受EGFR-TKIs治疗的糖尿病合并NSCLC患者的疗效，可以发现二甲双胍的使用与患者死亡风险的降低呈现显著相关，也就是说，二甲双胍的使用能显著提高药物治疗效果，降低患者死亡率。此外，相较于单独二甲双胍给药，联合EGFR-TKIs给药的患者呈现更长的中位PFS和中位OS。因此，EGFR-TKIs用于糖尿病合并肺癌的患者时，二甲双胍有可能成为首选口服降糖药。NCT01864681（CGMT）^[35]^是一项以双盲、随机和安慰剂对照的方式对既往未经治疗的携带EGFR突变的NSCLC患者的二甲双胍与安慰剂联合第一代EGFR-TKIs吉非替尼的疗效开展的II期临床研究。值得注意的是，研究发现在未患糖尿病的EGFR突变NSCLC患者中，联合二甲双胍治疗虽然没有明显增加毒性和加重患者的病情，但增加了腹泻的风险。因此，该研究不支持联合使用二甲双胍和EGFR-TKIs治疗未合并糖尿病的EGFR突变NSCLC患者。Chen等^[36]^通过用EGFR-TKIs加上二甲双胍和其他候选降血糖药物联合治疗患有糖尿病的NSCLC患者，观察患者的预后情况。研究发现二甲双胍组患者的PFS和OS明显长于其他候选降糖药组，而且ORR和疾病控制率（disease control rate, DCR）也显著高于其他候选降糖药组。这项研究揭示了二甲双胍和EGFR-TKIs在治疗携带EGFR激活突变的糖尿病合并NSCLC患者中具有协同作用，并且使用二甲双胍可以提高患者生存率和延迟或克服患者EGFR-TKIs获得性耐药。Han等^[37]^为了揭示二甲双胍对晚期NSCLC患者具有良好的疗效，进行了一项关于接受单独EGFR-TKIs和联合二甲双胍治疗的晚期NSCLC糖尿病患者临床病理特征和治疗结果的评估。该研究随机分配了具有EGFR突变的糖尿病合并肺腺癌患者，分别接受单独EGFR-TKIs和联合盐酸二甲双胍治疗，结果发现与单独EGFR-TKIs治疗组相比，联合二甲双胍治疗组的中位PFS和中位OS均显著延长。由于该研究的对象仅集中于服用二甲双胍的糖尿病患者，所以不能用于评价二甲双胍在非糖尿病患者中的作用。因此，后续还需要在更大规模的病例人群中做更多的调查来证实该研究的结论。为了进一步评估EGFR-TKIs与二甲双胍联合治疗具有EGFR突变的NSCLC患者的疗效和安全性，一项墨西哥国家癌症研究所发起的METLUNG项目已于2021年7月正式启动（NCT05445791）。该项目通过将具有EGFR突变的晚期NSCLC患者随机分配，分成使用EGFR-TKIs单药治疗与联合二甲双胍治疗组，并通过患者PFS和OS的评估，对EGFR-TKIs单药治疗与二甲双胍联合使用的疗效和安全性进行研究。虽然该项目目前还处于进展阶段，尚未公布其试验数据，但是该项目所进行的探索性研究对EGFR-TKIs联合二甲双胍治疗NSCLC策略的进一步完善提供了新的思路。此外，该项目在联合用药方面对临床实践的指导有很多值得探讨之处，有待深入挖掘。

综上，临床研究结果表明EGFR-TKIs单药和联合二甲双胍治疗相比，联合治疗可以更有效地延长NSCLC患者中位PFS和中位OS，更显著地改善患者的预后，但是在实际应用中，联合治疗需要选择适合的特定人群或者优化给药顺序才能够达到更好的治疗效果。因此，后续还需要进一步的临床研究以确定EGFR-TKIs联合二甲双胍治疗的最佳人群，使患者有更好的临床获益。此外，二甲双胍联合不同种类EGFR-TKIs治疗NSCLC可能存在疗效差异，如二甲双胍和第三代EGFR-TKIs奥希替尼联合治疗EGFR-TKIs获得性耐药的NSCLC具有协同作用，可以提高患者的中位OS长达8个月^[38]^，而与第一代EGFR-TKIs吉非替尼联用可以显著延长一线EGFR-TKIs获得性耐药的患者中位PFS长达6个月。除此之外，二甲双胍的剂量选择应该以美国食品药品监督管理局的要求为参考，做到安全、有效、合理和规范^[39]^。因此，对于要使用二甲双胍和EGFR-TKIs联合治疗的NSCLC患者，肿瘤诊疗方案的制定一定是个体化、充分考虑疗效差异和患者情况的；并且在考虑其疗效的同时，也要考虑是否存在不良反应和可能出现并发症的情况，尽量为患者制定最优的治疗方案。尽管目前已有部分研究证实EGFR-TKIs联合二甲双胍治疗NSCLC患者具有一定的疗效，但仍无法提供安全且有效的手段来应对EGFR-TKIs获得性耐药的现状，因此需要更多大样本、多中心的临床研究来进一步探讨联合治疗对NSCLC患者的有效性和安全性；其次，二甲双胍协同EGFR-TKIs靶向药物抵抗NSCLC、延缓EGFR-TKIs获得性耐药的机制有待进一步探索，为未来肺癌分子靶向治疗提供有力的辅助治疗方案。

## 5 小结与展望

根据以往的研究，EGFR-TKIs药物对EGFR阳性突变型NSCLC具有良好的治疗效果，但患者在后期仍要面临EGFR-TKIs获得性耐药的困境，因此EGFR-TKIs耐药是EGFR阳性突变型NSCLC患者治疗中的难题。目前的研究已经表明二甲双胍通过抑制EMT和IGF-1R、IL-6、TGF-β等信号通路克服NSCLC细胞对EGFR-TKIs耐药，而二甲双胍在与EGFR-TKIs联合应用中，调控LKB1/AMPK/TOR等通路抑制肿瘤细胞生长可为EGFR突变型NSCLC患者带来更好的PFS和OS。因此，二甲双胍和EGFR-TKIs联合用药方式可以给EGFR突变型NSCLC患者的治疗带来全新的思考。

尽管二甲双胍与EGFR-TKIs联用对EGFR突变型NSCLC患者疗效较佳，我们也要警惕联合用药引起的不良反应，如腹泻、胃肠道反应、低血糖等。因此为了避免或减少联合用药产生的不良反应，我们需要慎重控制二甲双胍的用法用量，或者采用其他相关的药物进行干预。此外，为更好地改善晚期NSCLC患者的不良预后问题，对于其治疗方案的选择，特别是新型靶向药物的联合治疗模式和细分人群的选择，还需要进一步的临床试验。

**Competing interests**

The authors declare that they have no competing interests.
